# Regional Downregulation of Dopamine Receptor D1 in Bilateral Dorsal Lateral Geniculate Nucleus of Monocular Form-Deprived Amblyopia Models

**DOI:** 10.3389/fnins.2022.861529

**Published:** 2022-06-08

**Authors:** Dongyue Lin, Zhonghao Wang, Wei Chen, Tao Shen, Xuan Qiu, Kun Wei, Jiahui Li, Dongsheng Yang, Ping Wang, Xuri Li, Jianhua Yan, Zhongshu Tang

**Affiliations:** ^1^State Key Laboratory of Ophthalmology, Zhongshan Ophthalmic Center, Guangdong Provincial Key Laboratory of Ophthalmology and Visual Science, Sun Yat-sen University, Guangzhou, China; ^2^Jinan Purui Eye Hospital, Children’s Eye Disease and Ocular Motor Institute of Purui Jinan, Jinan, China; ^3^Guangdong Province Key Laboratory of Brain Function and Disease, Zhongshan School of Medicine, Sun Yat-sen University, Guangzhou, China

**Keywords:** monocular deprivation, amblyopia, RNA sequencing, DRD1, mouse, monkey, LGN

## Abstract

Amblyopia is a common eye disease characterized by impaired best-corrected visual acuity. It starts in early childhood and leads to permanent vision reduction if left untreated. Even though many young patients with amblyopia are well treated in clinical practice, the underlying mechanism remains to be elucidated, which limits not only our understanding of this disease but also the therapeutic approach. To investigate the molecular mechanism of amblyopia, primate and rodent models of monocular-deprived amblyopia were created for mRNA screening and confirmation. We obtained 818 differentially expressed genes from the dorsal lateral geniculate nucleus (dLGN) of a primate model of amblyopia. After Gene Ontology and kyoto encyclopedia of genes and genomes (KEGG) enrichment analyses, the main enriched pathways were related to neural development. Interestingly, a particular neurotransmitter pathway, the dopaminergic pathway, was identified. The downregulation of dopamine receptor D1 (*DRD1*) was confirmed in both monkey and mouse samples. Furthermore, the immunofluorescence staining indicated that DRD1 expression was downregulated in both ventrolateral region of the contralateral dLGN and the dorsomedial region of the ipsilateral dLGN in the mouse model. The regions with downregulated expression of DRD1 were the downstream targets of the visual projection from the amblyopic eye. This study suggested that the downregulation of DRD1 in the LGN may be a cause for amblyopia. This may also be a reason for the failure of some clinical cases of levodopa combined with carbidopa applied to amblyopes.

## Introduction

Amblyopia is an ophthalmic disease that is diagnosed when the best-corrected visual acuity is lower than that of normal people at corresponding age, under the condition that there are no physical lesions in the eyes (Barrett et al., 2013). It is a common visual impairment and affects approximately 2–5% of the total population (Wang et al., 2020). In clinical practice, amblyopia is one of the most common causes of vision loss, not only in children but also in adolescents (Pediatric Eye Disease Investigator Group, 2004; Fronius et al., 2009). It is widely accepted that amblyopia develops during early childhood and vision loss will be permanent if it is left untreated (Leguire et al., 1993b). In addition, amblyopia may also lead to the reduction of a wide range of visual functions, such as stereoacuity, contrast sensitivity, and sensitivity to shape changes (Barrett et al., 2013).

Amblyopia can be caused by many conditions that prevent normal visual stimulation in childhood (Barrett et al., 2013). The most common conditions are anisometropia, strabismus, high ametropia, and form deprivation. Many animal models of amblyopia are indeed based on monocular form deprivation (Oray et al., 2004; Cheng et al., 2008). Clinically, amblyopia can occur in one or both eyes, but most cases are unilateral (Jefferis et al., 2015).

Fortunately, amblyopia can be well treated in clinical practice. A popular treatment is monocular occlusion, that is, covering the dominant eye with a patch (Giaschi et al., 2015; Jefferis et al., 2015; Maconachie et al., 2016). Monocular patching is very effective for young individuals with monocular amblyopia. In addition, the drug treatments are supplementary to patch treatment (Stryker and Lowel, 2018; Papageorgiou et al., 2019; Vagge et al., 2020). Of them, levodopa combined with carbidopa has been under study for years and acts by increasing the dopamine level in both cerebrum and retina (Ofori et al., 1986; Gottlob et al., 1989; Lewitt, 2016; Papageorgiou et al., 2019). This approach has been reported to improve vision over patching alone in some cases (Sengpiel, 2014); however, the outcome is not certain (Sengpiel, 2014).

Although young individuals with amblyopia are well treated, the underlying mechanism remains largely unknown. This is possible because of the complicated system involved in this disease. Although amblyopia is generally considered an eye disease, it is rooted in the brain (Chen et al., 2014). Defects in any region in the central visual system or in the connection between these regions may lead to this disease. The lack of knowledge of amblyopia limits not only our understanding of this disease but also the therapeutic approach. To explore the etiology of amblyopia at the molecular level, we started with mRNA screening of the central visual system of a monkey model of amblyopia and then with a mouse model for further confirmation and further study.

## Materials and Methods

### Establishment of Amblyopia Models

The experimental protocols were approved by the Animal Ethics Committee of the Zhongshan Ophthalmic Center, Sun Yat-sen University. All experimental procedures involving animals adhered to the Association Research in Vision and Ophthalmology. Generally, one eye of each animal was sutured during the critical period of vision development, and kept till the critical period ended.

To establish the primate model of amblyopia, 1-month-old baby cynomolgus monkeys were examined under optical coherence tomography, electroretinogram, fundus photography, and A-ultrasound to ensure that both eyes were well-developed, and there were no organic or functional defects in the eyes. After the examination, the left eye was sutured. Three months later, the monkeys were euthanized, and the dLGNs were isolated for RNA sequencing.

To establish the rodent model of amblyopia, the left eyes of postnatal day 18 C57/Bl6 mice were sutured. Twenty-four days later, the total LGN was isolated for mRNA and protein sample preparation. Alternatively, brain sections were prepared for immunofluorescence staining.

### The RNA Extraction, Library Construction and Sequencing

Total RNA was extracted using a Trizol reagent kit (Invitrogen, Carlsbad, CA, United States). The RNA quality was assessed on an Agilent 2100 Bioanalyzer (Agilent Technologies, Palo Alto, CA, United States) and checked using RNase free agarose gel electrophoresis. The mRNA was enriched by Oligo -deoxythymine beads, and processed for fragmentation and reverse transcription into cDNA with random primers. Second-strand cDNAs were synthesized and the cDNA fragments were purified, end repaired, A base added, and ligated to Illumina sequencing adapters. The ligation products were size selected by agarose gel electrophoresis, PCR amplified, and sequenced using Illumina Novaseq6000 by Gene Denovo Biotechnology Co., (Guangzhou, China).

### Bioinformatics Analysis

After removing the low-quality reads by fastp (version 0.18.0) (Chen et al., 2018), the clean reads were aligned with the reference genome [Macaca_fascicularis_5.0 in National Center for Biotechnology Information (NCBI) database] using HISA T2 (version 2.1.0) (Kim et al., 2015). Transcripts were reconstructed with software Stringtie (version 1.3.4) and their abundances were quantified by software StringTie in a reference-based approach (Pertea et al., 2015, 2016). The data are available in the Gene Expression Omnibus database GSE189488.

Gene Ontology (GO) enrichment analysis was performed for the functional classification of candidate genes (Ashburner et al., 2000). Pathway-based analysis was performed using the KEGG database (Kanehisa and Goto, 2000).

### Quantitative Polymerase Chain Reaction

Quantitative polymerase chain reaction was performed to quantify the relative expression level of genes as previously described (Zhang et al., 2017). The monkey and mouse primers are listed in Tables 1, 2, respectively.

**TABLE 1:** List of primers for qPCR of monkey samples.

Gene	Forward (5′-3′)	Reverse (5′-3′)
*GABRB2*	GTCACACGGCGAGAAAACAGG	GCACGGCGTACCAAAACATCA
*SSTR3*	CACCCTATGGGCAGGCAAAT	CAAGGAGGCATTCTCGGGTT
*DRD1*	CCACAGCGTTTCAGAGCCGT	CAATCCTGCGGACTGTCACTCTT
*ROBO1*	CTGGATGACTGTGGTGGTTGATA	TGCCTTTGTGATGATGCTTCC
*ROBO2*	GGAGTGGCAGTCTCTCAGGTG	GCACCAAGGCCTCCTCATC
*GRIN1*	GAGGAAGAACCTGCAGAGCACC	CCCTATGACGGGAACACAGCT

**TABLE 2:** List of primers for qPCR of mouse samples.

Gene	Forward (5′-3′)	Reverse (5′-3′)
*Gabrb2*	CCCCAACTAGACGGACTACCA	CTCCTCAGGCGACTTTTCTTT
*Robo2*	GGAGTGGCAGTCTCTCAGGTG	GAGCTAAAGGCTTTTCCT GTGAC
*Grin1*	GGTGGCTGGAGGCATCGT	TGGGATGGTACTGCTGCAGGT
*Sstr3*	ACATCTTCACAAGTCTGCCCAC	CCAAGGTCGTAGGCTCAGATG
*Robo1*	CCGCTACTTTGACAGTTCAA GAGC	CAGGACCTTGCCGAATGACT
*Drd1*	AGAAGTGACTCTAAAGCAAGG GCAT	TCCTGGTCAATCTCAGTCAC TTTTC

### Brain Section Preparation

Mice were transcardially perfused with cold saline for 2 min followed by 4% paraformaldehyde (PFA) for 30 min. Brains were isolated and immersed in 4% PFA solution for 24 h. The fixed brains were dehydrated in 20%, followed by 30% sucrose solution till they sank down to the bottom of the containers, and then embedded in optimal cutting temperature compound (Tissue-Tek, United States), cut into 30-μm thick frozen sections.

### Immunofluorescence Staining

Immunofluorescence staining was performed as described previously (Zhou et al., 2019). Briefly, brain sections were air-dried for 30 min at room temperature (RT), rinsed in 0.01 M phosphate buffered saline (PBS), and permeabilized with PBS supplemented with 0.5% Triton X-100 and 5% goat serum for 15 min at RT. Then, the sections were incubated in the following order: Primary antibodies against DRD1 (GeneTex, GTX100355) overnight at 4°C, Alexa Fluor 488-conjugated secondary antibody for 1 h at RT, and 0.1% DAPI for 5 min at RT, with washing in PBS 3 times after each incubation. The sections were examined under a confocal microscope (Nikon Eclipse Ni-E).

### Immunofluorescence Density Measurement

The immunofluorescence density measurement was carried out in accordance with the previous studies (Hanke, 2000; Torborg and Feller, 2004; Ogawa et al., 2005; Liu et al., 2020). For a semiquantitative estimation of DRD1 in the dLGN, a coronal section was selected in the middle of dLGN that was close to Figure 50 of The Mouse Brain atlas (Paxinos and Franklin, 2001). The images were captured after the laser intensity and gain were optimized in each channel. The boundary of the dLGN was identified and tracked with dotted lines. A large rectangle was drawn within the core dLGN along its long axis (from dorsomedial to ventrolateral), which was further divided into six areas, each corresponding to a region of interest (ROI). For the measurement, the background of each picture was adjusted to be consistent across blinded investigators. The fluorescence intensity of a neighboring region in the hippocampus was considered a control to be consistent in all samples. The color images were converted into binary images in ImageJ software (National Institutes of Health, Bethesda, MD, United States). The threshold percentage used for the conversion was also determined by the blinded investigators to ensure that the threshold percent value best represented the observed signal. The average fluorescence density was obtained by ImageJ.

### Statistical Analysis

Statistical analysis was carried out using the SPSS 20.0 statistical software package. All data were tested for normality and homogeneity of variance. The data with a normal distribution and homogeneity of variance were expressed as the mean and standard deviation (SD). Single factor analysis of variance (ANOVA) was used for comparison among groups and minimum significant difference (least significant difference, LSD). The *t*-test was used for pairwise comparisons between the groups. The difference was set to be statistically significant when *p* < 0.05.

## Results

### Visual Deprivation Leads to the Regulation of Neural Developmental Factors in Primate Dorsal Lateral Geniculate Nucleus

**FIGURE 1:**
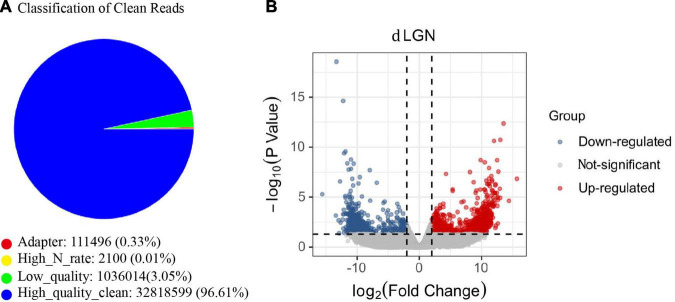
The mRNA sequencing of the dLGN of a primate model of monocular deprivation amblyopia. **(A)** Quality control of mRNA sequence. The proportion of all types of reads in the sample test. “Adaptor” indicates the proportion of the number of reads containing junction sequences to the total number of reads; “High_N_rate” indicates the proportion of the number of reads containing unknown bases to the total number of reads; “Low_quality” indicates the proportion of the number of low-quality reads to the total number of reads. “High_quality_clean” is the proportion of the original sequence data to the total number of reads after removing impurities. **(B)** The volcano plot of the fold change and statistical significance. Gray points in the plot represent mRNA with no significant differences. Red points represent upregulated mRNAs, and blue points represent downregulated mRNAs with statistical significance.

To explore the etiology of amblyopia, we established a primate model of monocular visual deprivation by suturing the left eyes of baby monkeys. Three months later, brain regions involved in the central visual system were isolated for RNA sequencing. Here, we followed the mRNA sequencing results of the dorsal lateral geniculate nucleus (dLGN). Three samples from the untreated control monkeys and two samples from the ipsilateral (left) dLGN of amblyopia monkeys were qualified for sequencing (Figure 1A). We identified 818 differentially expressed genes (DEGs) that were regulated more than 2 times (Figure 1B). Of them, 492 were upregulated, and 326 were downregulated.

**FIGURE 2:**
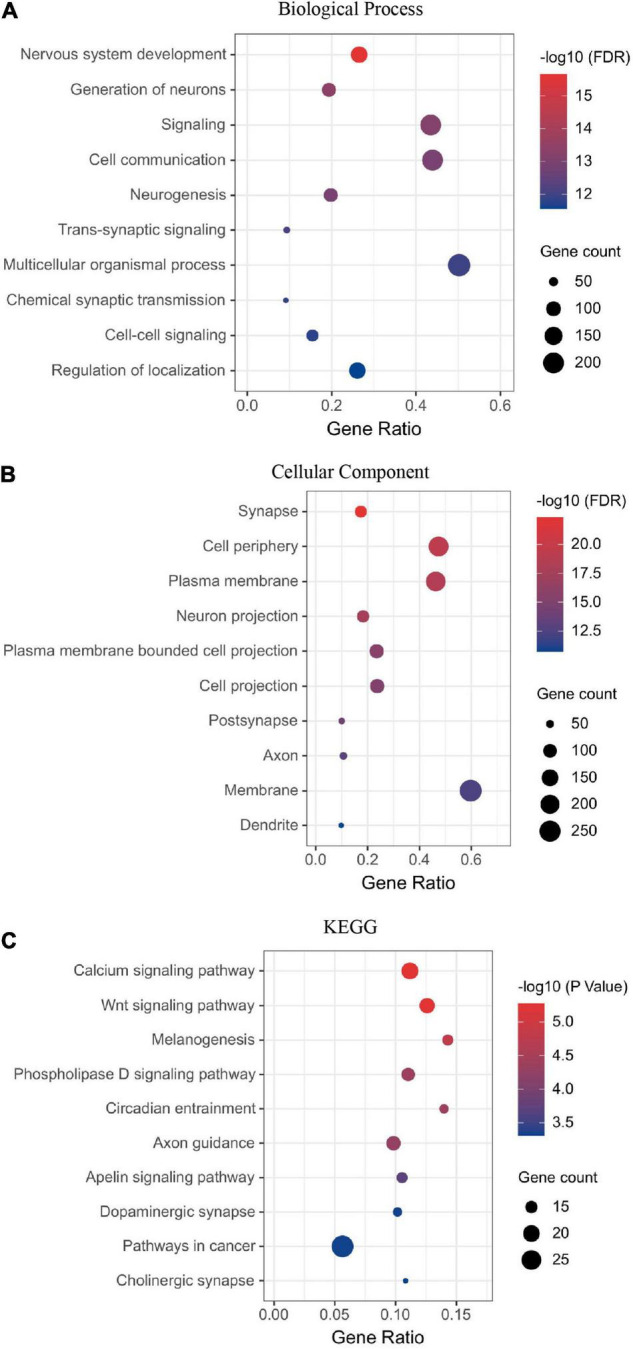
Visual deprivation leads to the regulation of neural developmental factors in the primate dLGN, as shown in the enrichment analysis of DEGs **(A)** Biological process. **(B)** Cellular component. **(C)** KEGG analysis of DEGs.

Next, we performed the enrichment analysis. The GO enrichment demonstrated that the most enriched categories were related to nervous system development. In the biological process (BP) category, many DEGs were related to cell–cell signaling or communication, neurogenesis, and neural development (Figure 2A). In the cell component (CC) category, many DEGs were related to synapse, membrane, and projection (Figure 2B). The KEGG enrichment analyses identified pathways related to synapse, nervous system development, and neurogenesis (Figure 2C). Thirteen DEGs were grouped in the dopaminergic pathway (Figure 2C). Dopamine receptor D1 (DRD1) is a member of this pathway. A list of DEGs classified by a biological process is available in the supplementary.

Thus, the mRNA sequencing results demonstrated that visual deprivation leads to the regulation of neural developmental factors in the monkey dLGN.

### Dopamine Receptor D1 Is Downregulated in the Lateral Geniculate Nucleus of Amblyopia Models

**FIGURE 3:**
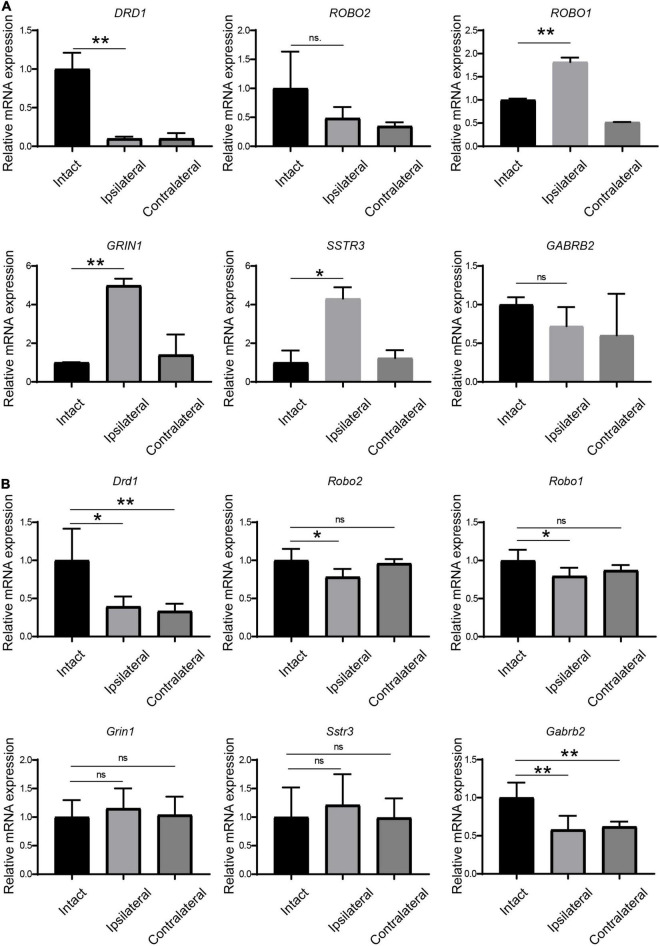
Verification of the regulation of receptors in LGN samples from amblyopia models by qPCR. **(A)** Verification in the monkey model of amblyopia. **(B)** Verification in the mouse model of amblyopia. Data represent the mean ± SE. *n* = 3 for **(A)** and *n* = 5 for **(B)** in all groups, *p < 0.05, **p < 0.01.

Because synaptic receptors play pivotal roles in neural plasticity and functions, we selected six receptors from the DEG list for further verification (Table 3). First, we checked the expression of these receptors in monkey LGN by qPCR. The up- or downregulation of these receptors was verified to be the same as the results from mRNA sequencing (Figure 3A).

**TABLE 3:** Receptors selected for verification that were associated with neural development.

ID	Gene symbol	Intact count	Ipsilateral* count	Intact_ fpkm	Ipsilateral* fpkm	log2 (FC)	*p* value
XM_005558557.2	*DRD1*	2283.8	391.1	34.5	6.6	−2.4	0.003619547
XM_005567378.2	*SSTR3*	41.8	272.7	0.6	2.9	2.3	0.045261342
XM_015446591.1	*ROBO1*	26.7	219.3	0.3	1.8	2.7	0.024738274
XM_015445436.1	*ROBO2*	530.9	1.9	3.7	0.0	−8.0	1.9956E-08
XM_005580340.2	*GRIN1*	45.5	453.2	0.8	5.6	2.8	0.019992267
XM_005558445.2	*GABRB2*	227.6	0.0	2.1	0.0	−11.0	0.000460176

*“Ipsilateral” means the dLGNs on the side ipsilateral to the sutured eyes.

Next, we examined the expression of these receptors by qPCR in amblyopia mouse samples. As shown in Figure 3B, *Drd1* and *Gabrb2* were downregulated in both ipsi- and contralateral LGNs in the mouse model, which was consistent with the corresponding regulation in the monkey model. The other four selected receptors were not regulated in the same ways as they were in the monkey model.

### Dopamine Receptor D1 Is Specifically Downregulated in the Target Regions of Mouse Amblyopic Eyes

**FIGURE 4:**
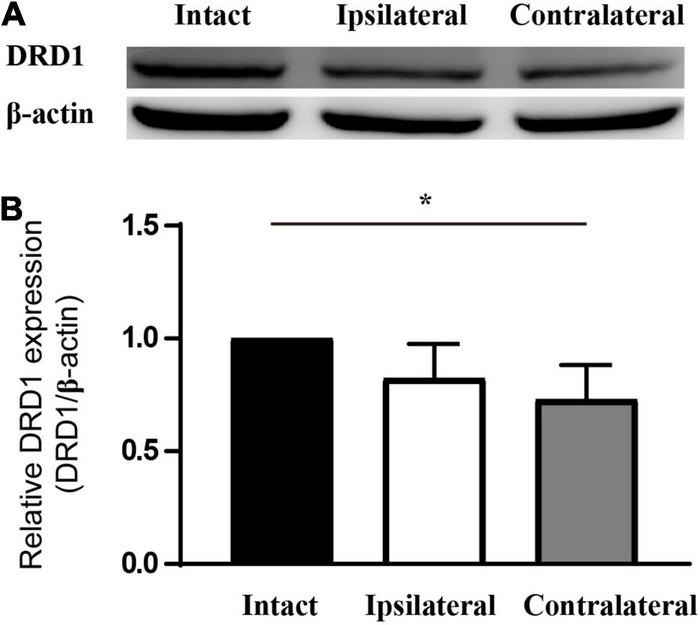
DRD1 is downregulated in the LGN of monocularly deprived amblyopic mice. **(A)** Western blot of samples from mouse LGN. **(B)** Statistics of the relative expression level of DRD1. Set the value of intact LGN as 1. Data represent the mean ± SE. *n* = 3, *p < 0.05.

Of the six receptors under investigation thus far, the regulation of *Drd1* was consistent in mRNA sequencing and in the qPCR study. In addition, we know that levodopa, a precursor of dopamine, has been under clinical trials for the treatment of amblyopia for years. Therefore, *Drd1* was selected for further investigation. Thus, we checked the protein expression of DRD1 in the LGN of the mouse amblyopia model by Western blot. The semiquantitative study showed a 15–20% downregulation of expression in both ipsilateral and contralateral LGNs (Figure 4).

**FIGURE 5:**
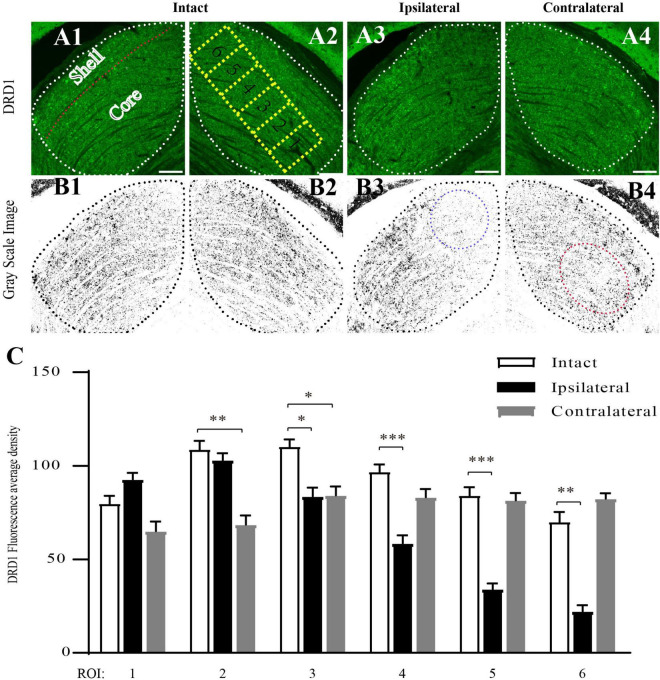
Downregulation of DRD1 in dLGNs in the target regions of mouse amblyopic eyes. **(A1–A4)** Anti-DRD1 immunostaining in the dLGN. The dashed white line delineates the dLGN. The shell and core regions are separated with a dashed red line in **(A1)**. Six ROIs (region of interest) in **(A2)** represent the areas for measuring and statistics. **(B1–B4)** Grayscale image of anti-DRD1 immunostaining for statistics. The most downregulated areas are indicated with a dashed blue circle in **(B3)** and a dashed red circle in **(B4)**. **(C)** Quantification of the normalized fluorescence intensity of DRD1 staining. Data represent the mean ± SE, *n* = 8 for the intact group, *n* = 6 for both ipsilateral and contralateral groups, *p < 0.05, **p < 0.01, ***p < 0.001.

Next, we examined the distribution of DRD1 in the subregions of the mouse dLGN by immunostaining. In the coronal sections of intact (control) mouse brains, DRD1 immunoreactive (IR) cells and fibers were identified in the dLGN. A strong IR was detected in the outer shell portion and the ventrolateral region of the inner core portion. Light IR was detected in the dorsomedial region of the inner core portion (Figures 5A1–A2,B1–B2).

The pattern of DRD1 IR in the dLGN of the amblyopic mice was similar to that in the control group. However, lighter staining was identified in the dorsomedial region of the ipsilateral dLGN (Figures 5A3,B3) and in the ventrolateral region of the contralateral dLGN (Figures 5A4,B4) compared to the intact group.

To obtain a semiquantitative estimation of the intensity of DRD1 staining, we analyzed its regional expression. Green DRD1 IR was converted to gray. The core portion of the dLGN was divided into six rectangles along its long axis, each corresponding to a region of interest (ROI). The average staining intensity in each ROI was calculated. As shown in Figure 5C, in the ipsilateral dLGN, DRD1 IR was at a similar level as that of the control in ROIs 1–3 but was downregulated to less than half of the level of the control group in ROIs 5–6. In the contralateral dLGN, DRD1 IR was downregulated to approximately two-third of the level of the control group in ROIs 1–3 but not in ROIs 4–6. Both regions of downregulation were exactly the targets of the projections from the amblyopic eye. These data demonstrated the downregulation of DRD1 in the dLGN in the target regions of the amblyopic eye.

## Discussion

In the mRNA sequencing study of the dLGN from the primate model of amblyopia, we obtained over 800 DEGs. The most enriched categories of these DEGs were related to neural development. Of these DEGs, *DRD1* was confirmed to be downregulated in both primate and rodent amblyopia models. More interestingly, an immunofluorescence study revealed that it was specifically downregulated in bilateral dLGNs in the target region of the visual projection from the form-deprived eye.

The qPCR study demonstrated that the regulation of the selected receptors was not the same pattern between the monkey and mouse models. The difference may be caused by many reasons. The first is the different ratios of crossed and uncrossed retinal projections into the LGN. In the mouse optic nerve, approximately 90% of retinal axons cross the midline and project to the contralateral LGN, with less than 10% projecting to the ipsilateral LGN. However, in the primate optic nerve, the ratio of ipsi- vs. contralateral projection is 1:1 (Herrera and Mason, 2007). The second reason may be the different structures of the LGN between primates and rodents. The primate LGN exhibits a laminar structure, whereas the rodent LGN is essentially homogeneous (Seabrook et al., 2017). A third reason is that the two models were closed at different time points. The monkey model was closed at 4 months when the critical period is still on (Daw, 1998). The mouse model was closed after the end of the critical period (Huberman et al., 2008).

The significance of this study lies in four aspects. First, it provides an overall map of factors in the LGN which are regulated in amblyopia. Although it is well known that amblyopia is a developmental disease, the molecular mechanism is still unclear. The broad range of factors identified in this developmental disease delineates a large map of factors regulated in the dLGN of amblyopia animal models, although many of them are still to be confirmed and studied in detail.

Second, this study suggests a specific molecular mechanism of amblyopia, that is, the downregulation of DRD1 in the target region of the form-deprived eye. As a catecholamine, dopamine is an important neurotransmitter involved in neural function and plasticity (Kasamatsu and Pettigrew, 1979; Zhou et al., 2000; Guo et al., 2002). A decrease in dopamine was observed in the retina of both rat and monkey models of monocular deprivation and thus was considered a possible reason for amblyopia (Melamed et al., 1986; Imamura and Kasamatsu, 1989; Iuvone et al., 1989; Sengpiel, 2014). Taken together, our and the former findings suggest that impaired development of the DA system in the retina-dLGN pathway could be a molecular mechanism of amblyopia.

The third significance is that this study presents a possible reason for the failure of levodopa treatment for amblyopia. Levodopa has been in clinical trials of amblyopia treatment for over 30 years (Hoyt, 2015; Vagge et al., 2020). However, its effect is uncertain (Leguire et al., 1993a; Procianoy et al., 1999). The decrease in DRD1 in the LGN in the target region of the amblyopic eye provides one possible reason for the failure of some cases. As the main dopaminergic receptor, the regional decrease in DRD1 in the LGN may lead to the failure of levodopa application. On the other hand, this study also opens the possibility of novel strategies for amblyopia treatment, which means DRD1 in the dLGN can be another target for amblyopia treatment.

The fourth significance would be that we revealed LGN is also a highly impacted region in amblyopia. Over the past decade, the research into animal models and clinical cases has led to a growing understanding of the pathology of amblyopia. The primary and secondary visual cortices are often considered the basis of amblyopia (Hess, 2001; Hess et al., 2009, 2010, 2011; Brown et al., 2013). The LGN is less studied. As the primary recipient of the outputs from both eyes, the LGN is a site of precortical binocular processing (Dougherty et al., 2021). The significant role of the LGN lies in its locus between the retina and the visual cortex. Its special position suggests that it may play important roles in the formation of amblyopia (Wong et al., 2005; Baker et al., 2007; El-Shamayleh and Movshon, 2011).

Despite the significance drawn so far, we know that there are still some limitations in this study, which means more work to be done to understand the mechanism of amblyopia. First of all, we mainly followed DRD1 from the long list of candidates. It would be fascinating to verify and fellow the roles of more factors in the process of amblyopia formation. Even for DRD1, more works are still waiting, such as functional study and its relationship with other amblyopia factors in LGN.

Secondly, this study only focused on dLGN, more work is needed to verify the sequencing results of the other brain regions. An intriguing study would be to investigate the relationship between the amblyopia factors in LGN and other regions.

The third limitation would be that it is still an open question whether the downregulation of DRD1 is a primary cause of amblyopia or just a downstream consequence of monocular form deprivation. We may determine that the initial downregulation of DRD1 in both LGNs could be caused by the failure of vision stimulation from the form-deprived eye because the form deprivation is the initial treatment in our animal models. However, the downregulation of DRD1 leads to more downstream defects. Thus, it should be investigated in clinical trials.

In summary, we identified the downregulation of DRD1 in dLGN in the target regions of form-deprived eye. It suggests DRD1 as an amblyopia factor. Its regional downregulation may be the reason for the failure of levodopa in the treatment for amblyopia. Also, DRD1 itself may serve as a new target of clinical trials.

## Data Availability Statement

The datasets presented in this study can be found in online repositories. The names of the repository/repositories and accession number(s) can be found in the article/Supplementary Material.

## Ethics Statement

The animal study was reviewed and approved by the Animal Ethics Committee of the Zhongshan Ophthalmic Center, Sun Yat-sen University.

## Author Contributions

ZW, DY, PW, XL, JY, and ZT conceived this study. DL, ZW, WC, TS, and XQ performed the experiments. DL, KW, WC, and JL analyzed the data and prepared the figures. DL and ZT wrote the manuscript. DY, PW, JY, and XL reviewed and edited the manuscript. All authors contributed to the article and approved the submitted version.

## Conflict of Interest

The authors declare that the research was conducted in the absence of any commercial or financial relationships that could be construed as a potential conflict of interest.

## Publisher’s Note

All claims expressed in this article are solely those of the authors and do not necessarily represent those of their affiliated organizations, or those of the publisher, the editors and the reviewers. Any product that may be evaluated in this article, or claim that may be made by its manufacturer, is not guaranteed or endorsed by the publisher.
